# Simultaneous quantification of multiple specific food allergen proteins indicates varied allergen content in diagnostic and therapeutic preparations

**DOI:** 10.1186/2045-7022-5-S3-P29

**Published:** 2015-03-30

**Authors:** Anna Kuklinska-Pijanka, Elisabeth Young, Martin Chapman, James Hindley

**Affiliations:** 1 Indoor Biotechnologies ltd, Cardiff, United Kingdom

## 

In order to help allergic patients manage often severe symptoms, food manufacturers are required to list allergens on labels whereas researchers are working to improve diagnostics and develop effective immunotherapies. Existing tools using generic immunoassays do not provide precise identification or quantification of specific food allergens. Our goal was to develop an accurate, sensitive and high throughput assay that would enable simultaneous quantification of multiple allergens in foods and in allergen preparations used for oral food immunotherapy (OIT).

Fluorescent beads coupled to allergen specific monoclonal antibodies were used to develop a multiplex array for simultaneous quantification of major allergens from peanut (Ara h 1, Ara h 2, Ara h 6), milk (Bos d 5), egg (Gal d 1), and shrimp tropomyosin. Target allergens were detected using biotinylated antibodies and streptavidin conjugated fluorochrome. The array was quantified using highly purified natural allergens as standards. Allergen content was measured in NIST Standard Reference Material (SRM) peanut butter, diagnostic extracts, and extracts used for OIT. The results were compared to ELISA.

There was a high correlation between the multiplex array and allergen specific ELISA (R2= 0.82 to 0.98). The sensitivity of the array was increased by up to 40-fold compared to ELISA, demonstrating accuracy as low as 20 pg/ml. Significant differences were found between specific peanut allergen levels (Ara h1: Ara h 2: Ara h 6 ratio) in NIST SRM peanut butter (4:1:1) compared with peanut flour extracts used for OIT (1:3:3) and diagnostic peanut extract (1:5:6).

In conclusion, an accurate and sensitive multiplex array for major food allergens was developed, revealing the varied allergen content of diagnostic and therapeutic preparations. The flexibility of the microsphere technology allows for further expansion to produce a comprehensive array for the most important food allergens. Ultimately, this quantitative multiplex array will enable more effective assessment of the potency of food extracts used for immunotherapy and allergy diagnosis and may also help to reduce the risk of inadvertent contamination of food.

